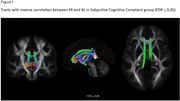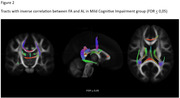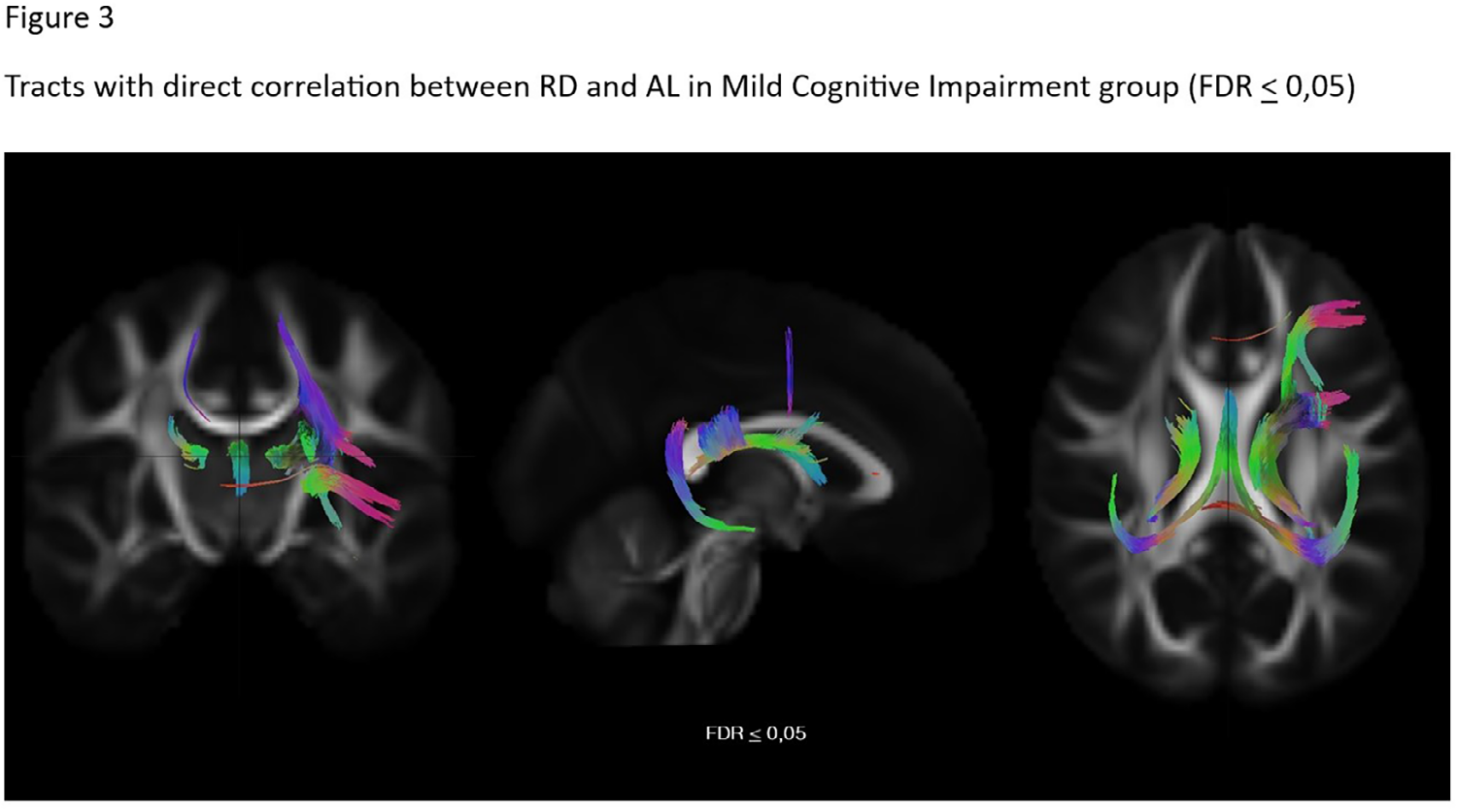# Correlations between white matter microintegrity and risk level of allostatic load in cognitive complaint subjects

**DOI:** 10.1002/alz.085867

**Published:** 2025-01-09

**Authors:** Patricio Riquelme Contreras, Fernando Henriquez, Claudio Román Godoy, Ingrid Buller‐Peralta, Cecilia Gonzalez Campo, Daniela Thumala, Patricia Lillo, Cecilia Okuma, Michele Demanet, María Francisca Damm, Pamela Guevara Alvez, El‐Deredy Wael, Graciela Muniz‐Terrera, Andrea Slachevsky Chonchol

**Affiliations:** ^1^ Geroscience Center for Brain Health and Metabolism (GERO), Santiago Chile; ^2^ Neuropsychology and Clinical Neuroscience Laboratory (LANNEC), Physiopathology Department ‐ ICBM, Neuroscience and East Neuroscience Departments, Faculty of Medicine, Universidad de Chile, Santiago Chile; ^3^ Memory and Neuropsychiatric Center (CMYN), Neurology Department, Hospital del Salvador and Faculty of Medicine, Universidad de Chile, Santiago Chile; ^4^ Department of Medical Technology. Faculty of Medicine. Universidad de Chile, Santiago Chile; ^5^ Masters in biological sciences / Neurosciences. Universidad de Valparaíso, Chile., Valparaíso Chile; ^6^ Interdisciplinary Center for Neuroscience (NeuroUC) ‐ Laboratory for Cognitive and Evolutionary Neuroscience ‐ Medicine School ‐ Pontificia Universidad Católica de Chile, Santiago Chile; ^7^ Memory and Neuropsychiatric Center (CMYN), Neurology Service, Hospital del Salvador and Faculty of Medicine, Universidad de Chile, Santiago Chile; ^8^ Universidad de Chile, Santiago Chile; ^9^ Geroscience center for mental health and metabolism, Santiago de Chile, Metropolitana Chile; ^10^ Health Engineering I&D Center. Universidad de Valparaíso., Valparaíso, Valparaíso Chile; ^11^ University of Edinburgh, Edinburgh, Edinburgh UK; ^12^ CONICET, Buenos Aires Argentina; ^13^ Cognitive Neuroscience Center (CNC), Universidad de San Andrés, Buenos Aires, Buenos Aires Argentina; ^14^ Laboratory of Neuropsychology and Clinical Neuroscience (LANNEC), Physiopathology Program‐ICBM, East Neurologic and Neurosciences Departments, Faculty of Medicine, University of Chile, Santiago Chile; ^15^ Department of Psychology, University of Chile, Santiago Chile; ^16^ Universidad de Chile, Santiago, Santiago Chile; ^17^ Instituto de Neurocirugía Dr. Alfonso Asenjo, Santiago Chile; ^18^ Geroscience center for mental health and metabolism (GERO), Santiago de Chile, Metropolitana Chile; ^19^ Memory and Neuropsychiatry Clinic (CMYN), Santiago de Chile, Metropolitana Chile; ^20^ Biomedical Engineering Department. Universidad de Concepción, Concepción, Bio Bio Chile; ^21^ Faculty of Engineering. Universidad de Valparaíso., Valparaíso, Valparaíso Chile; ^22^ Edinburgh Dementia Prevention, University of Edinburgh, Edinburgh UK; ^23^ Centre for Clinical Brain Sciences at the University of Edinburgh, Edinburgh UK; ^24^ Department of Social Medicine, Ohio University, Athens, OH USA; ^25^ University of Edinburgh, Edinburgh UK

## Abstract

**Background:**

Chronic exposition to stressor factors has been postulated as a cause of structural changes in the brain in the context of dementia. One of these changes can be the fiber integrity loss, that can be measured by diffusion tensor imaging (DTI). We obtained DTI whole brain metrics to relate them with allostatic load in subjects of a chilean cohort of cognitive complaint subjects.

**Method:**

We selected 135 subjects of a chilean cohort of cognitive complaint subjects and classified based in MoCA test in subjects with (58) (MCI) and without (77) (SCC) cognitive decline groups. To measure AL, we selected 15 metabolic, cardiovascular and immunitary biomarkers and the subjects were categorized in low, medium, and high‐risk categories of AL. DTI was calculated using a deterministic tractography algorithm and the relationships were established using a correlational tractoghraphy tool, based in DSIstudio, calculating the FDR (≤ 0,05) of whole brain fasciculus. Age and education was regressed out. After that, fasciculus with significance were segmented based on HCP 1065 atlas.

**Result:**

we obtained significative correlations in many fibers, depending on the parameter studied. Is interesting to note that bilateral cingulum bundles, and corpus callosum integrity are within the fibers that have relationship with allostatic load in both cognitive groups. Additionally, MCI group presents more compromised tracts than SCC group.

**Conclusion:**

In this study, a relationship between allostatic load and DTI integrity measurements in MCI and SCC groups was found. An interesting correlation exists in fornix and bilateral cingulum bundles, that can be supported by findings in previous studies based in human and animal models where the stress effects in white matter were studied. These results open an interesting research area to explore the role of stress response and mediators in early steps of dementia, who until today, has been poorly studied.